# Zinner Syndrome Unmasked by Workup for Renal Colic and Uncontrolled Hypertension

**DOI:** 10.7759/cureus.8381

**Published:** 2020-05-31

**Authors:** Amman Yousaf, Hafiz Muhammad Fazeel, Mohammed Hamza Shah, Fariha Ghaffar, Syeda Sabeeka Batool

**Affiliations:** 1 Radiology, Hamad General Hospital, Doha, QAT; 2 Radiology, Services Institute of Medical Sciences, Lahore, PAK; 3 Internal Medicine, Services Institute of Medical Sciences, Lahore, PAK; 4 Body Imaging/Radiology, Hamad Medical Corporation, Doha, QAT; 5 Internal Medicine, Allama Iqbal Medical College, Lahore, PAK; 6 Internal Medicine, University of Alabama at Birmingham Huntsville Regional Medical Center, Huntsville, USA

**Keywords:** renal agenesis, congenital defect, obstructive uropathy, seminal vesicle cyst, case report, renal colic

## Abstract

Zinner syndrome is a rare hereditary disorder of the mesonephric duct. The triad of the absence of one kidney, ipsilateral cystic dilatation of the seminal vesicle, and ejaculatory duct obstruction makes the diagnosis. Mostly, it is asymptomatic. However, genitourinary manifestations and workup for the incidental absence of one kidney often uncover the disease. Ultrasound and CT scan can identify the absence of a kidney and seminal vesicle cyst, while MRI is the gold standard for diagnostic elaboration of the pelvic anatomy. In this article, we have presented a 51-year-old male patient who presented with renal colic and hypertension. Radiological investigations for the renal colic uncovered the diagnosis of Zinner syndrome incidentally. This case highlights the incidental nature, variability in the clinical presentation, and the diagnostic challenges of this rare disorder. It also emphasizes on the radiologist for a careful evaluation of the pelvic images in patients with unilateral absence of a kidney.

## Introduction

Zinner syndrome (ZS) is a rare hereditary disorder characterized by a triad of unilateral renal agenesis, ipsilateral cystic dilatation of seminal vesicle, and ejaculatory duct obstruction [1]. First described by Zinner in 1914, it was initially considered as a mere manifestation of ureterocele [2]. To our knowledge, there are around 300 reported cases in the literature, with an estimated prevalence of less than 0.0001% [3]. Patients usually remain asymptomatic, but some may present in the second to fourth decades of life with a wide range of symptoms depending on the pelvic anatomy and the functional status of the contralateral kidney [1]. Treatment options depend on the severity of symptoms, the anatomy of the genitourinary tract, and the question of preservation of fertility. We present a case of a 51-year-old male patient who presented with new-onset uncontrolled hypertension, along with three-day history of left renal colic. As a part of the workup pertinent to his clinical presentation, imaging incidentally revealed the diagnostic features of ZS.

## Case presentation

A 51-year-old Filipino male patient presented to the emergency department with left-sided renal colic for two days. He had a family history of hypertension and was recently diagnosed with new-onset hypertension six months ago. The patient had a poor blood pressure control with amlodipine 10 mg and valsartan 160 mg. He also had a history of smoking and social drinking. On presentation, he was afebrile, and his blood pressure was 183/95 mmHg with a heart rate of 80 beats per minute, respiratory rate 17 breaths per minute, and SpO_2_ 99% on room air. Physical examination showed no significant findings except a mild tenderness in the left flank. Laboratory tests showed mild leukocytosis with an absolute neutrophilic count of 920 cells/µL, serum creatinine of 133 µmol/L, and a urea level of 7.50 mmol/L. Complete blood count, liver function tests, serum electrolytes, serum lactic acid, and urine dipstick were unremarkable.

**Figure 1:**
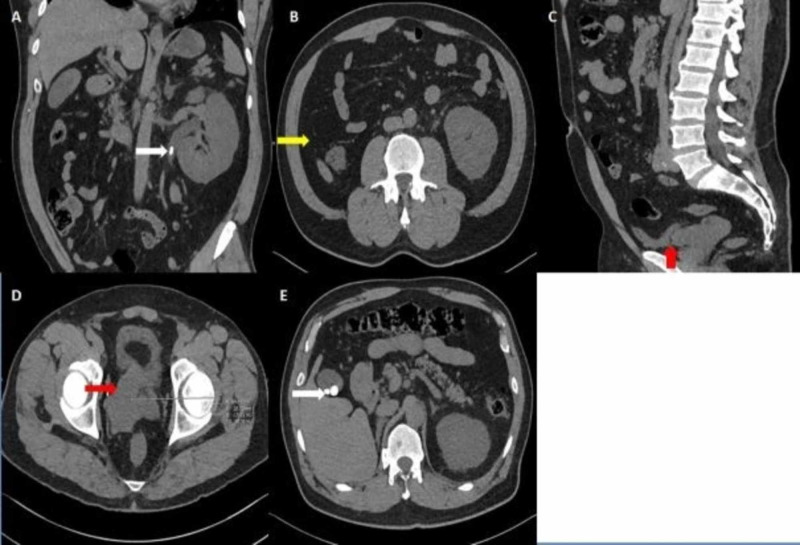
Unenhanced CT of the abdomen and pelvis An oblique coronal view demonstrates a left upper ureteric obstructing stone, causing dilatation of the proximal ureter and pelvicalyceal system (A). An axial section at the renal level portrays that the right renal fossa is empty, with no renal tissue or surgical materials (B). A sagittal and an axial cut revealing a right retrovesical possibly cystic lesion in the expected anatomic location of the right seminal vesicle (C, D). An axial cut of the upper abdomen exhibiting incidental finding of a few gallbladder stones without surrounding inflammatory changes (E).

The patient underwent an unenhanced CT scan of the abdomen and pelvis to rule out obstructive uropathy. It revealed an obstructing radiodense stone (CT density of 800 HU) in the left proximal ureter causing upstream hydroureteronephrosis. The right kidney and ureter were absent at their expected anatomical location in the abdomen and pelvis. Nevertheless, the radiologist found a retrovesical cystic mass (with a CT density of 18 HU), indenting the right posterior and right lateral wall of the urinary bladder, incidentally (Figure *1*).

**Figure 2:**
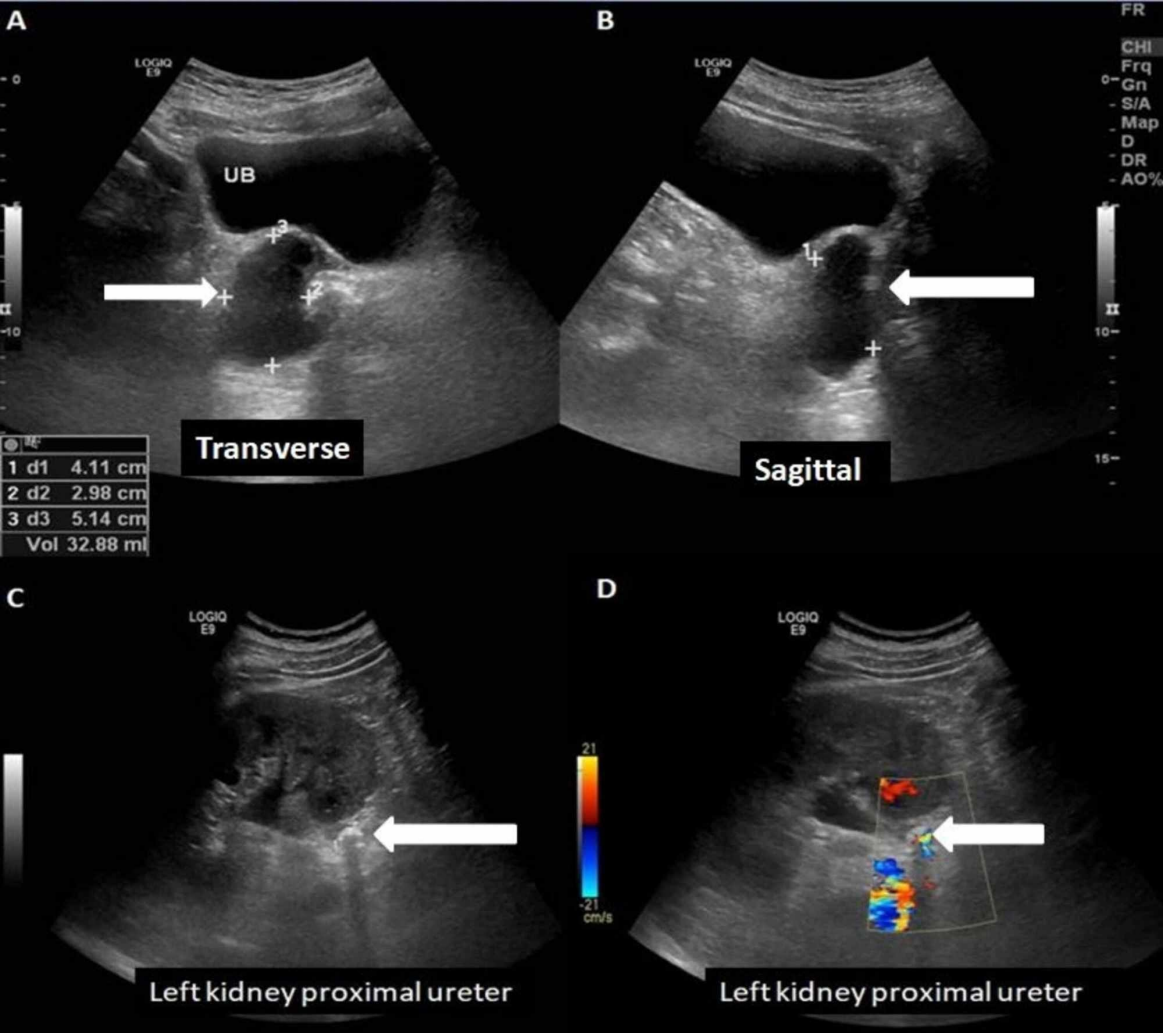
Ultrasound of the abdomen and pelvis A well-defined cystic lesion with faint internal echoes/debris, which is indenting the right posterolateral urinary bladder wall (A, B). A hyperechoic structure with posterior acoustic shadowing and twinkling artifact in the proximal ureter, associated with upstream pelvicalyceal dilatation, consistent with an obstructing stone (C, D).

Ultrasound examination confirmed its cystic nature (Figure *2*).

**Figure 3:**
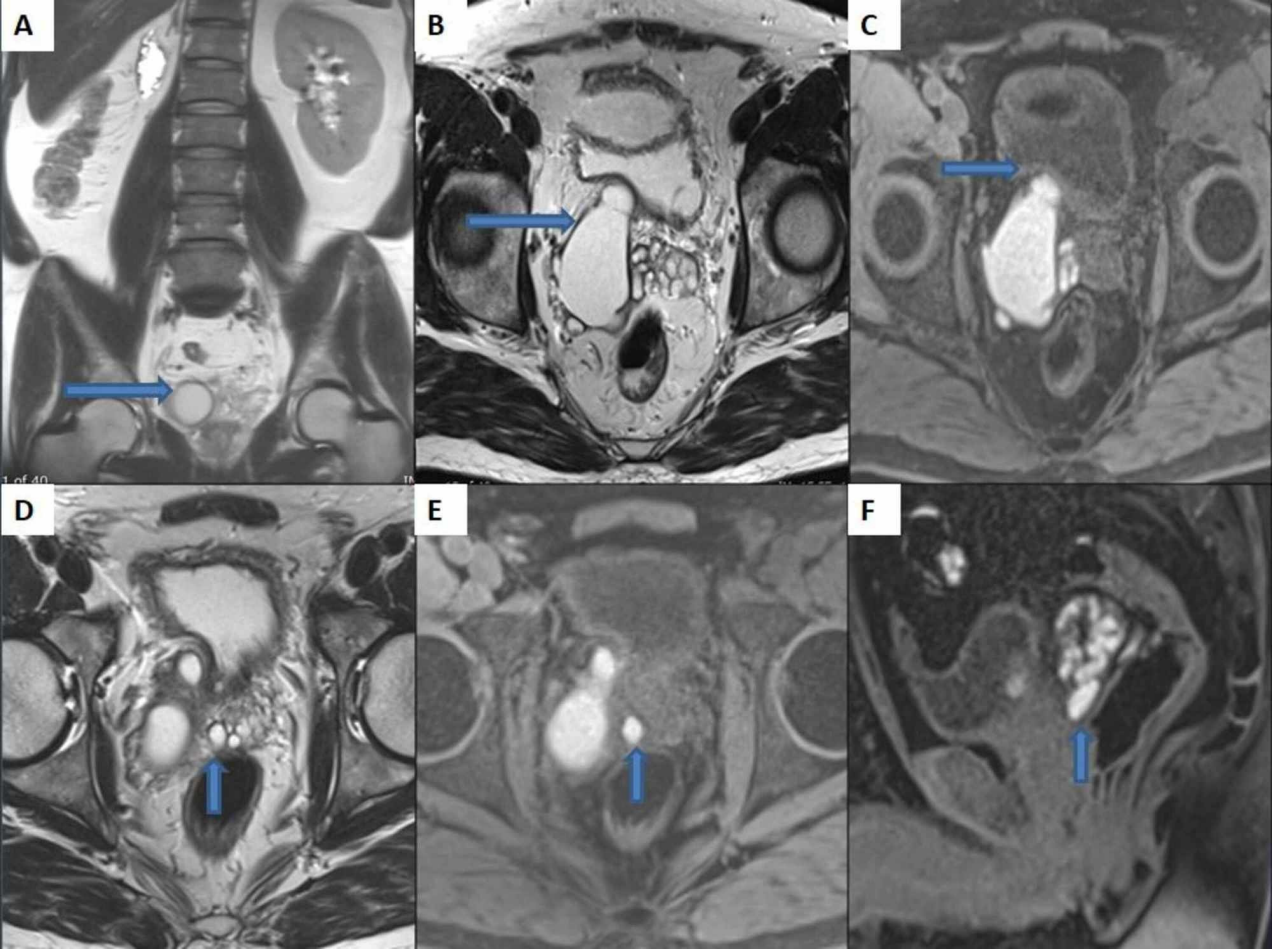
MRI of the pelvis Wide field of view coronal T2-weighted image (A) showing absent right kidney and a structure in the right hemipelvis with high T2 signal intensity (cystic). Axial T2 (B) and axial T1 fat-sat (C) images show cystic dilatation of right seminal vesicle (6 x 2.5 cm in anteroposterior and transverse dimensions, respectively) with T1-hyperintense hemorrhagic or proteinaceous content, which is indenting the right posterolateral urinary bladder wall. Axial T2 (D), axial T1 fat-sat (E), and sagittal T1 fat-sat (F) images show dilated right ejaculatory duct (especially the distal segment which measures 8 mm in diameter) along the right posterolateral aspect of the prostate. The contents of this cystic dilatation are similar to the dilated right seminal vesicle.

The patient underwent left cystourethroscopy for a double J stent placement. It did not reveal the orifice of the right ureter in the bladder. Further investigation with MRI pelvis showed cystic dilatation of the right seminal vesicle indenting the posterior wall of the urinary bladder. The described lesion showed a high intensity on T2 and T1, indicating proteinaceous or hemorrhagic fluid content. An 11-mm ejaculatory duct cyst was also identified (Figure *3*).

This triad of incidental findings made the diagnosis of ZS. As the patient had no complaints related to the seminal vesicle and ejaculatory duct cyst and had four children, he decided to have no further management for these cysts. The patient underwent two sessions of extracorporeal shock wave lithotripsy (ESWL) for the stone in the lower calyx of the left kidney. After two months, the stent was removed surgically, and cystourethroscopy was uneventful. The patient’s renal profile became normal, and blood pressure was controlled well with amlodipine 5 mg once daily. On a six-month follow-up, the patient had no complaint and maintained blood pressure control.

## Discussion

ZS is considered a sequela of the malformation of the mesonephric (Wolffian) duct. Malformation in the proximal segment of the mesonephric duct during the 13th to 14th weeks of gestation can lead to the growth failure of the ureteric bud, resulting in agenesis or dysgenesis of the kidney [4]. Likewise, a malformation in the distal mesonephric duct results in atresia of the ejaculatory duct, which may cause the chronic obstruction and cystic dilation of the seminal vesicles [4]. Mayer-Rokitansky-Kuster-Hauser (MRKH) syndrome is sometimes considered the female counterpart of this disorder, in which women develop uterine agenesis and malformed vagina [1]. However, external genitalia develop normally, and this correlation is debatable as the upper vagina originates from the Mullerian duct. A few cases in the literature demonstrate bilateral ejaculatory duct cysts as a part of ZS [5].

In most cases, the condition remains asymptomatic and diagnosed incidentally during the workup for unrelated conditions. However, the patient can present with a wide range of genitourinary manifestations like hematospermia, recurrent episodes of gross hematuria, ejaculation failure, and infertility. Other mild nonspecific symptoms include scrotal pain, perineal discomfort, and urinary frequency [1].

In patients presenting with genitourinary symptoms, ultrasound can be an initial imaging modality after the detailed clinical and family history. Ultrasound, however, may lead to misdiagnosis of the seminal vesicle cysts as varicocele [6]. CT scan and MRI are more reliable diagnostic modalities [1]. Nevertheless, in some reported cases, the CT scan proved insufficient for proper identification of the pelvic abnormalities [7]. MRI is the gold standard imaging modality because it can better delineate pelvic anatomy and malformations due to its high-resolution [7]. MRI depicts variable signal intensities depending upon the contents of the seminal vesicular cyst. Seminal vesicle cysts may show high signal intensity on T2-weighted images and intermediate signal intensity on T1-weighted images analogous to fluid, with negative gadolinium contrast enhancement. 

The severity of the symptoms and anatomical features can assist in deciding the treatment options. For asymptomatic patients, the observation by routine follow-ups is enough [8]. In patients with troublesome genitourinary symptoms, surgery is the standard approach. Open surgery using a transabdominal or transperineal approach has been the traditional choice. However, minimally invasive techniques are now the gold standard approach due to lesser blood loss, better outcomes, and shorter duration of hospitalization [9]. Recently, robotic-assisted interventions have shown to have curative potential in patients with long-standing local symptoms [10]. For patients with only symptoms related to seminal vesicle cysts, literature favors the transurethral unroofing as the treatment option [11]. Van den Ouden et al., while reporting data for 52 patients, demonstrated a 100% success rate of surgical excision of seminal vesicle cysts compared to a 75% success rate with transurethral unroofing [12]. However, the decision must be individualized depending on the patient's symptoms, anatomy, and preference. Cyst aspiration has a lower success rate (30%), providing only transient relief with high recurrence rates [12]. 

The question of fertility status and influence of syndrome on fertility in asymptomatic patients remains unanswered. The general reported approach in available literature favors surgery in symptomatic patients and advocates a thorough investigation of fertility status. The patients with fertility issues can have good outcomes from the assisted reproductive techniques [13]. Scientific data are lacking regarding the oncogenic potential of ZS for genitourinary tumors. Until now, the condition is considered benign, and there is no evidence of malignant transformation in the literature. However, these patients should follow the routine precautions, regular medical monitoring, and lifestyle modifications as advised for patients with one kidney.

## Conclusions

ZS is a rare genitourinary disorder characterized by a triad of unilateral renal agenesis, ipsilateral seminal vesicle cyst, and ipsilateral ejaculatory duct obstruction. Clinicians should be aware of the variable presentation pattern of this condition and should raise the suspicion in patients with incidental finding of an absent kidney.
